# Supplementary figures and images for: Differential miRNA and Protein Expression Reveals miR-1285, Its Targets TGM2 and CDH-1, as Well as CD166 and S100A13 as Potential New Biomarkers in Patients with Diabetes Mellitus and Pancreatic Adenocarcinoma
Source: Cancers (Basel). 2024 Jul 31;16(15):2726. doi: 10.3390/cancers16152726 (PMC11311671; doi:10.3390/cancers16152726)

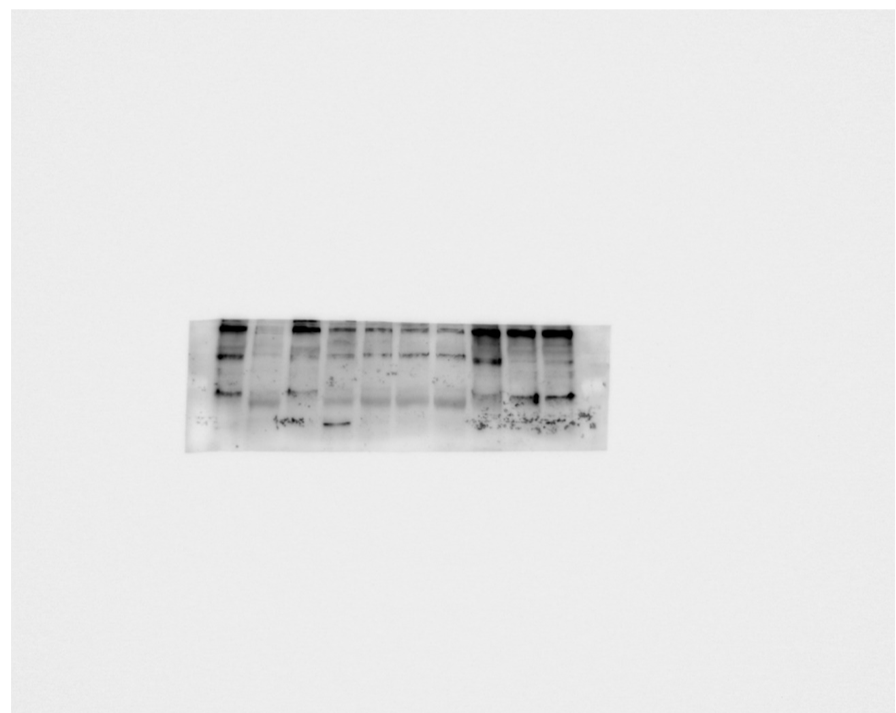

Supplement: Supplementary file 1 [file cancers-16-02726-s001.zip › WB data/CD166 PDAC+DM.tif]

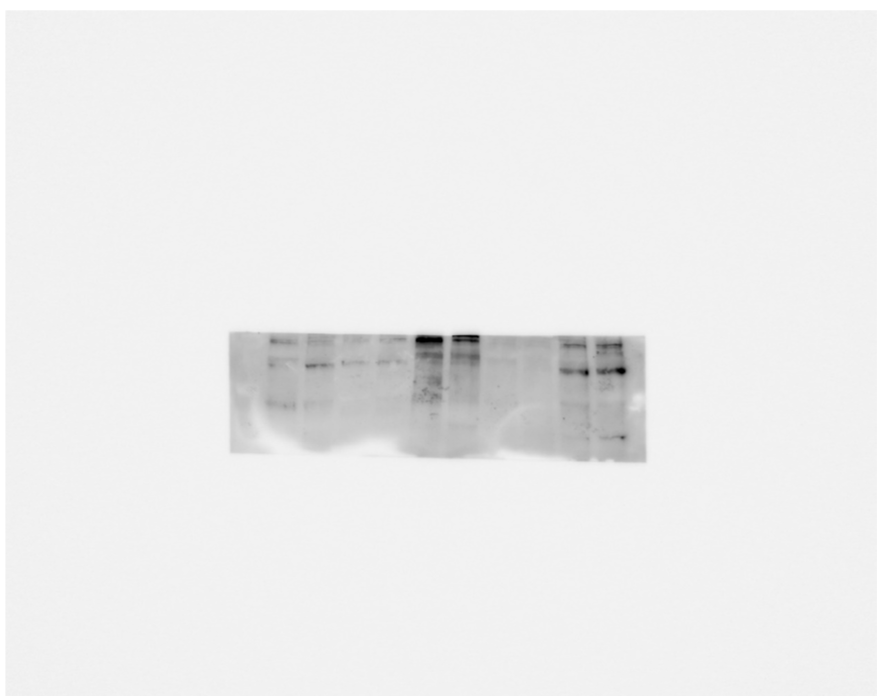

Supplement: Supplementary file 1 [file cancers-16-02726-s001.zip › WB data/CD166 PDAC.tif]

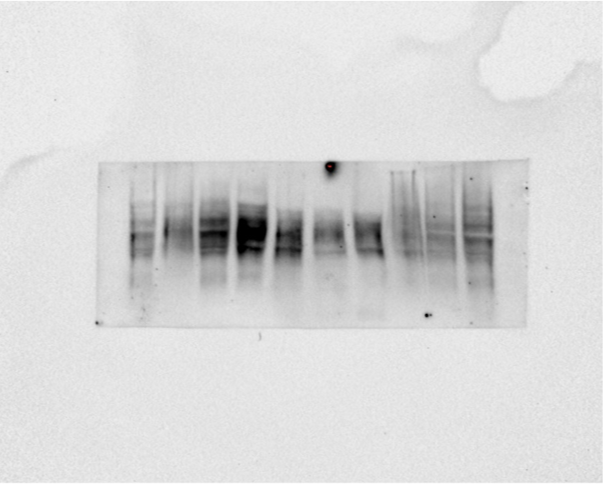

Supplement: Supplementary file 1 [file cancers-16-02726-s001.zip › WB data/CD63 PDAC+DM.tif]

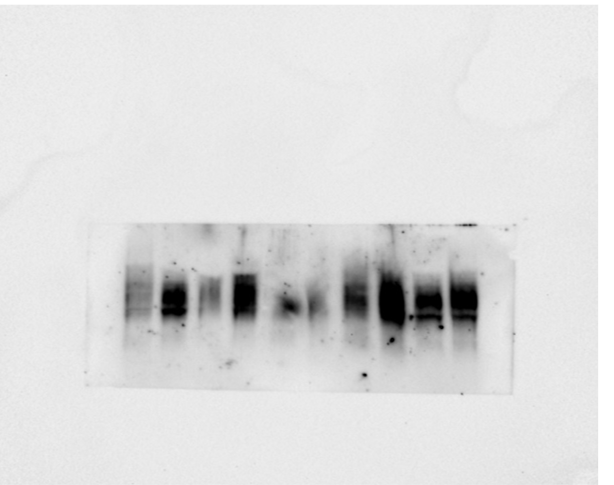

Supplement: Supplementary file 1 [file cancers-16-02726-s001.zip › WB data/CD63 PDAC.tif]

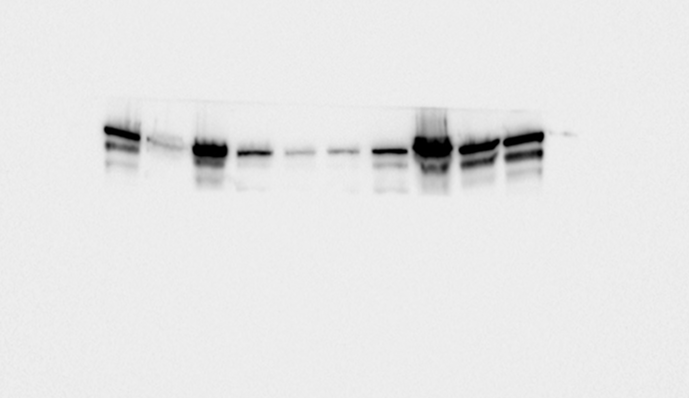

Supplement: Supplementary file 1 [file cancers-16-02726-s001.zip › WB data/CDH-1 PDAC + DM 1.tif]

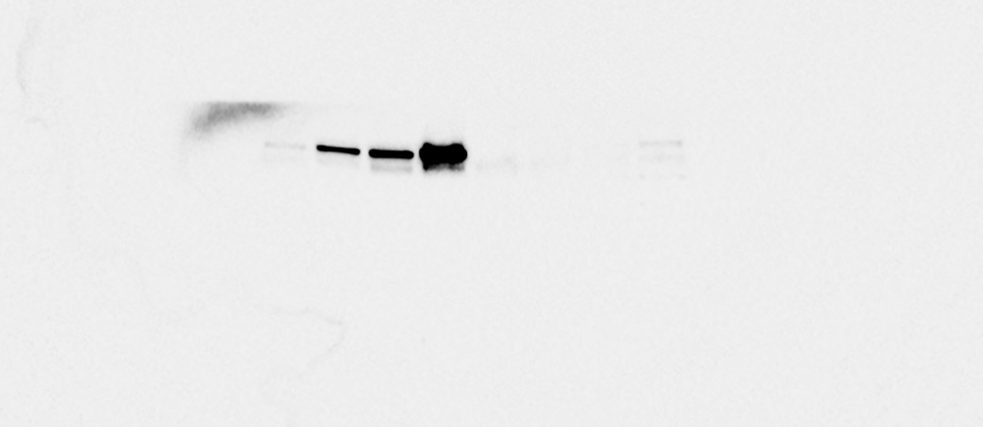

Supplement: Supplementary file 1 [file cancers-16-02726-s001.zip › WB data/CDH-1 PDAC + DM 2.tif]

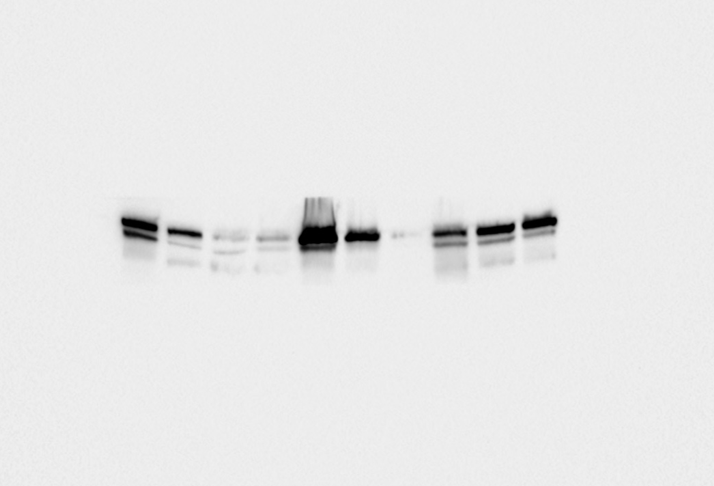

Supplement: Supplementary file 1 [file cancers-16-02726-s001.zip › WB data/CDH-1 PDAC.tif]

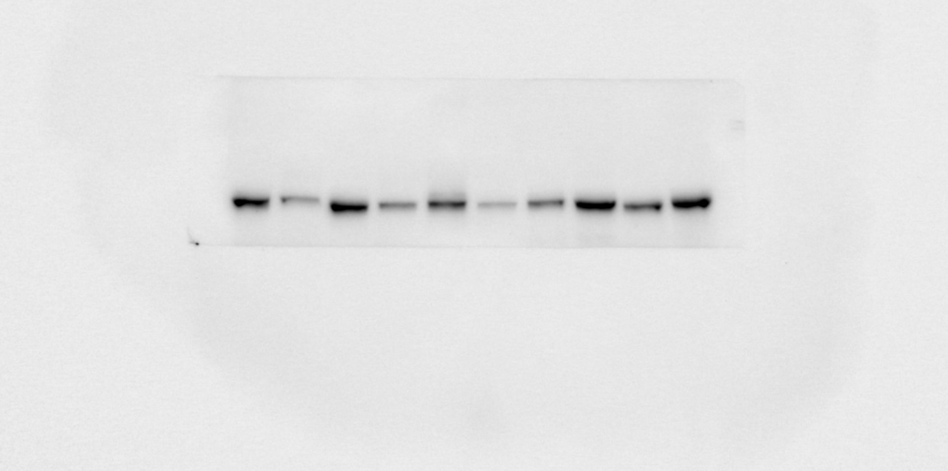

Supplement: Supplementary file 1 [file cancers-16-02726-s001.zip › WB data/cJUN PDAC+DM .tif]

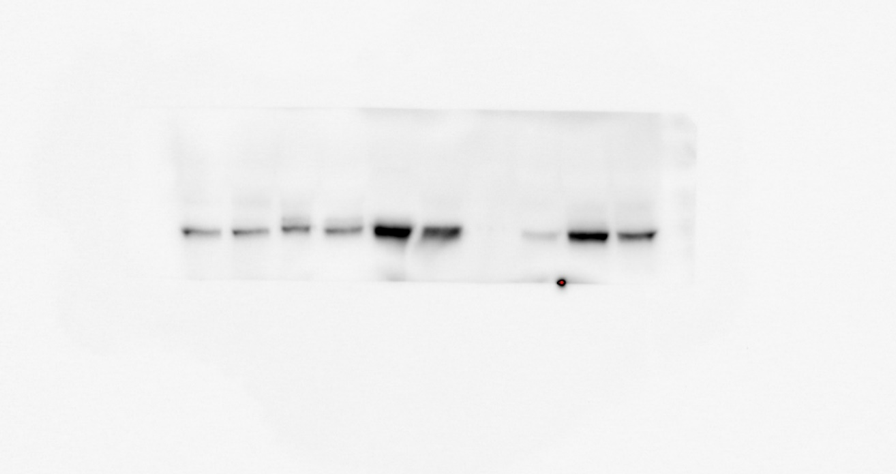

Supplement: Supplementary file 1 [file cancers-16-02726-s001.zip › WB data/cJUN PDAC.tif]

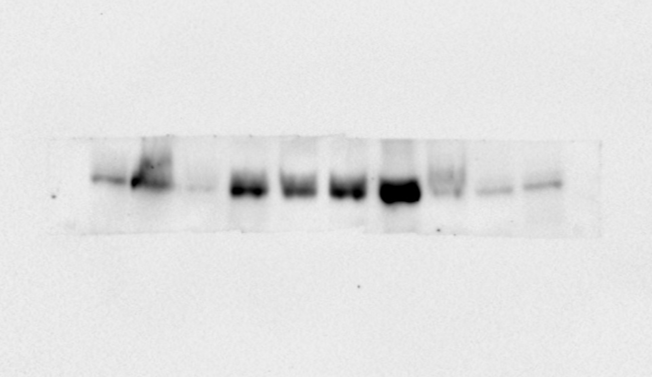

Supplement: Supplementary file 1 [file cancers-16-02726-s001.zip › WB data/p53 PDAC+DM.tif]

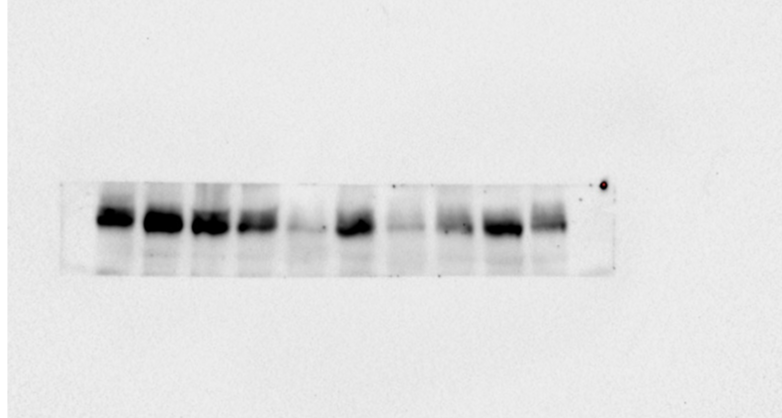

Supplement: Supplementary file 1 [file cancers-16-02726-s001.zip › WB data/p53 PDAC.tif]

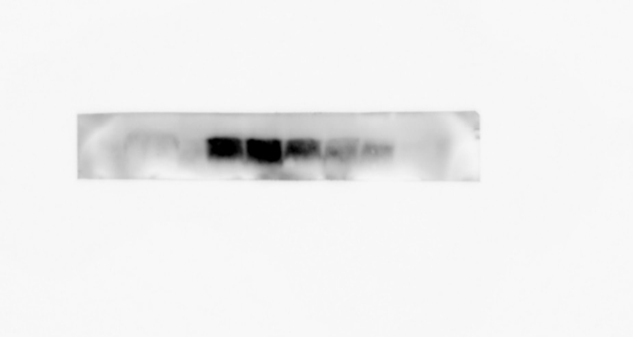

Supplement: Supplementary file 1 [file cancers-16-02726-s001.zip › WB data/S100A13 PDAC+DM 1.tif]

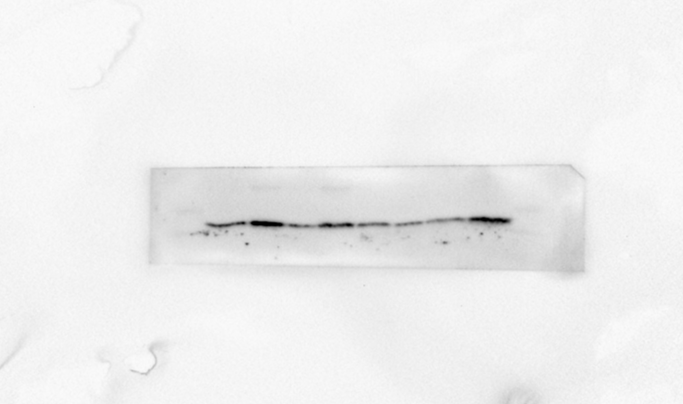

Supplement: Supplementary file 1 [file cancers-16-02726-s001.zip › WB data/S100A13 PDAC+DM 2.tif]

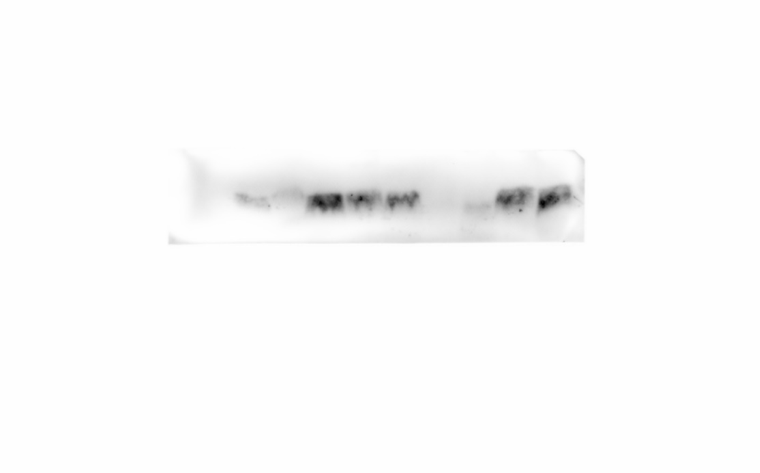

Supplement: Supplementary file 1 [file cancers-16-02726-s001.zip › WB data/S100A13 PDAC.tif]

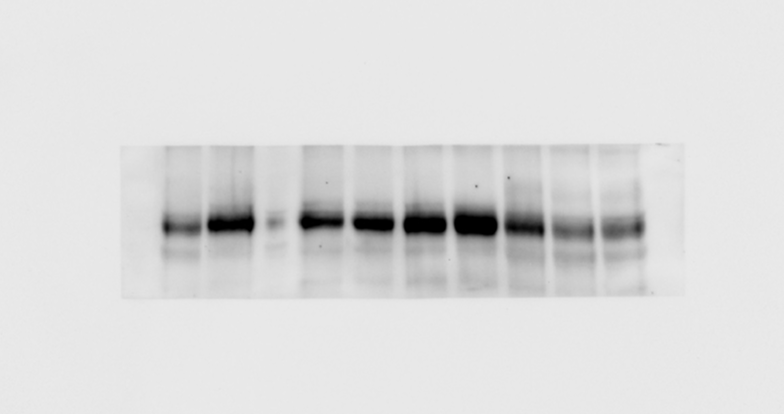

Supplement: Supplementary file 1 [file cancers-16-02726-s001.zip › WB data/smad 4 PDAC +DM 1.tif]

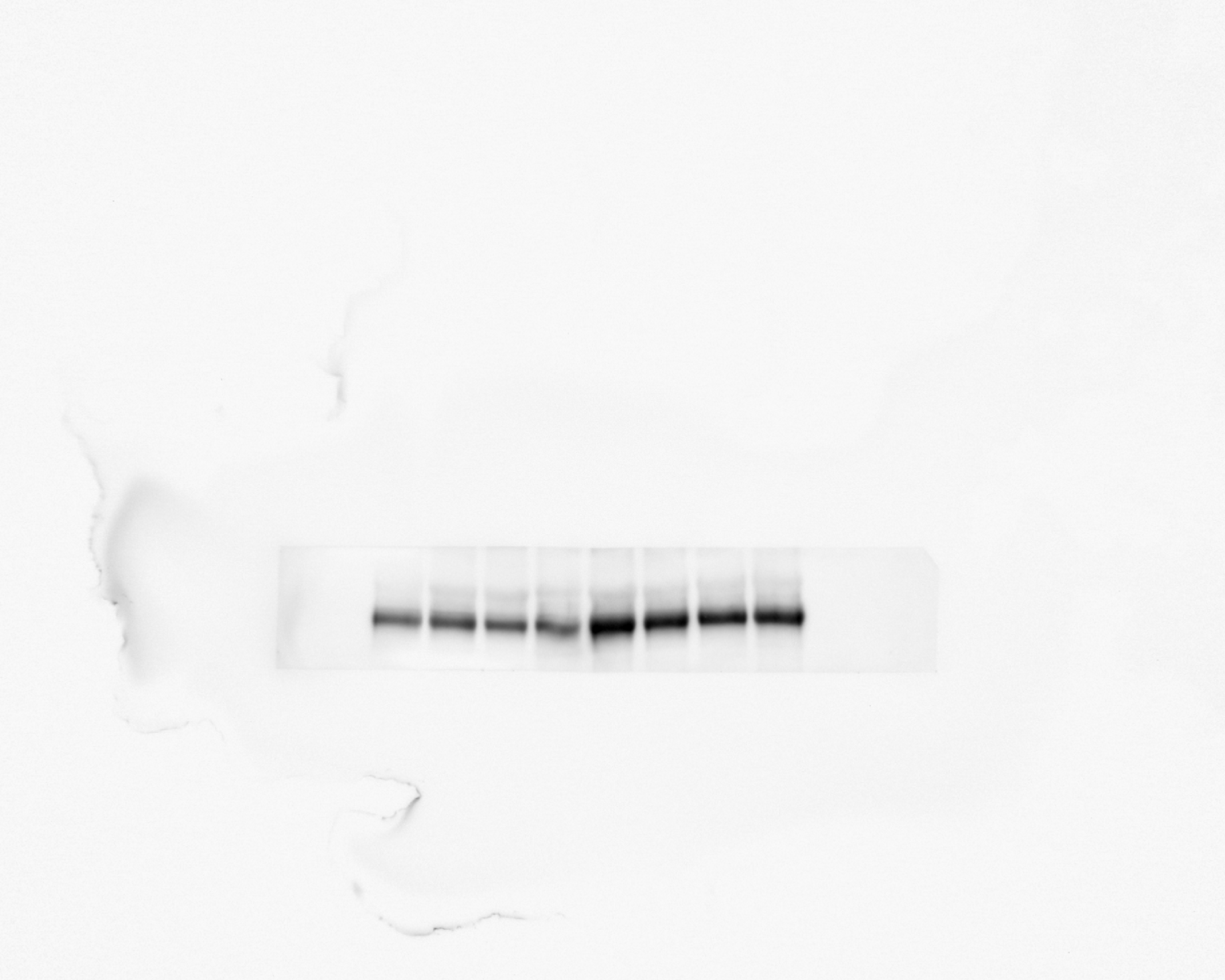

Supplement: Supplementary file 1 [file cancers-16-02726-s001.zip › WB data/Smad 4 PDAC +DM 2.tif]

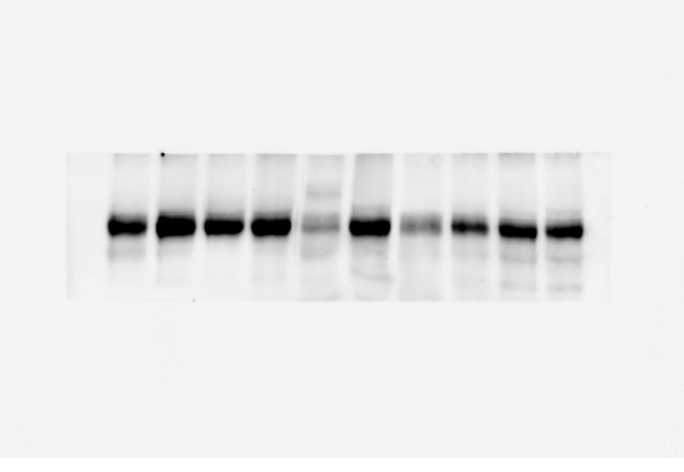

Supplement: Supplementary file 1 [file cancers-16-02726-s001.zip › WB data/smad 4 PDAC.tif]

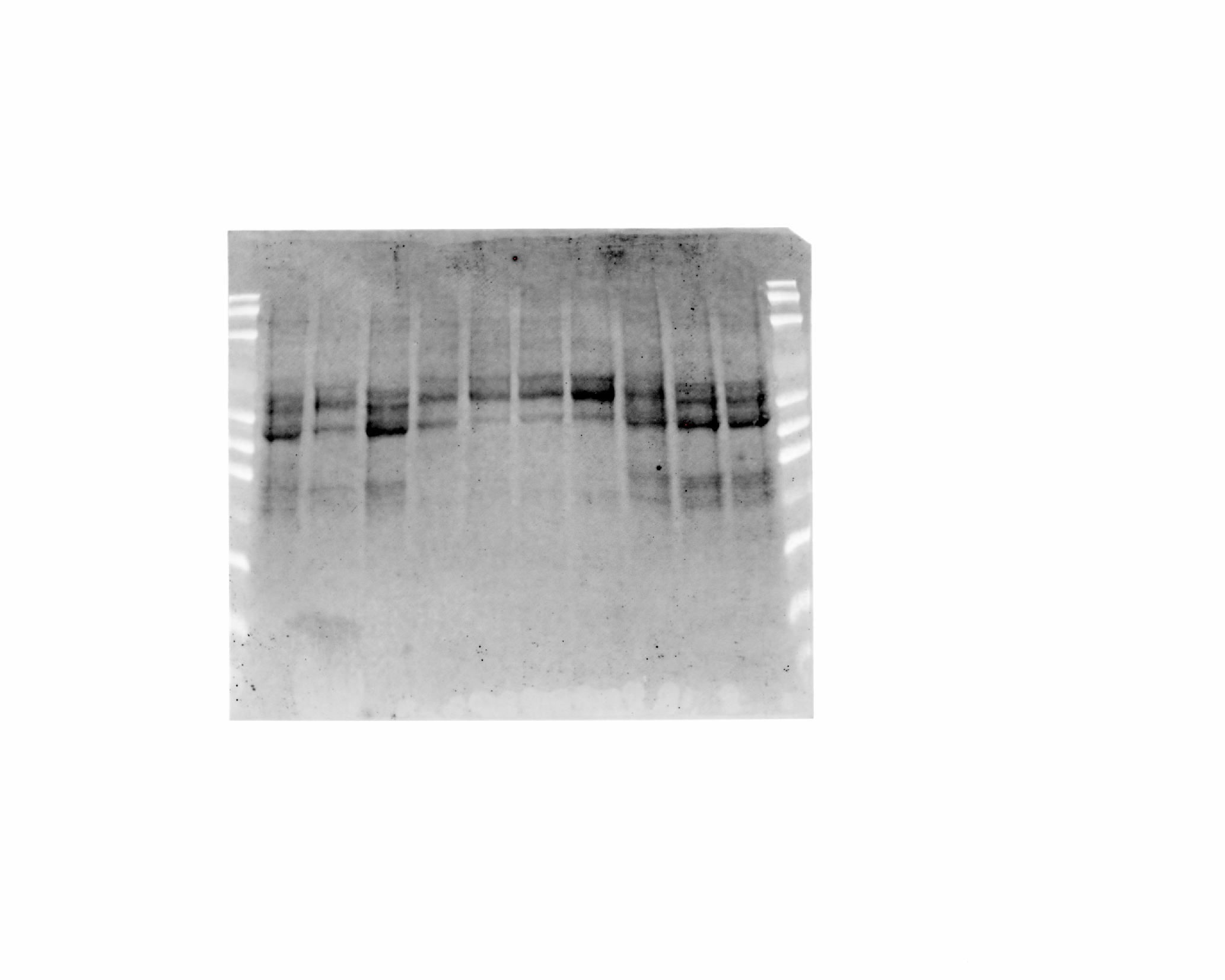

Supplement: Supplementary file 1 [file cancers-16-02726-s001.zip › WB data/Stain Free Blot CD166 PDAC+DM.tif]

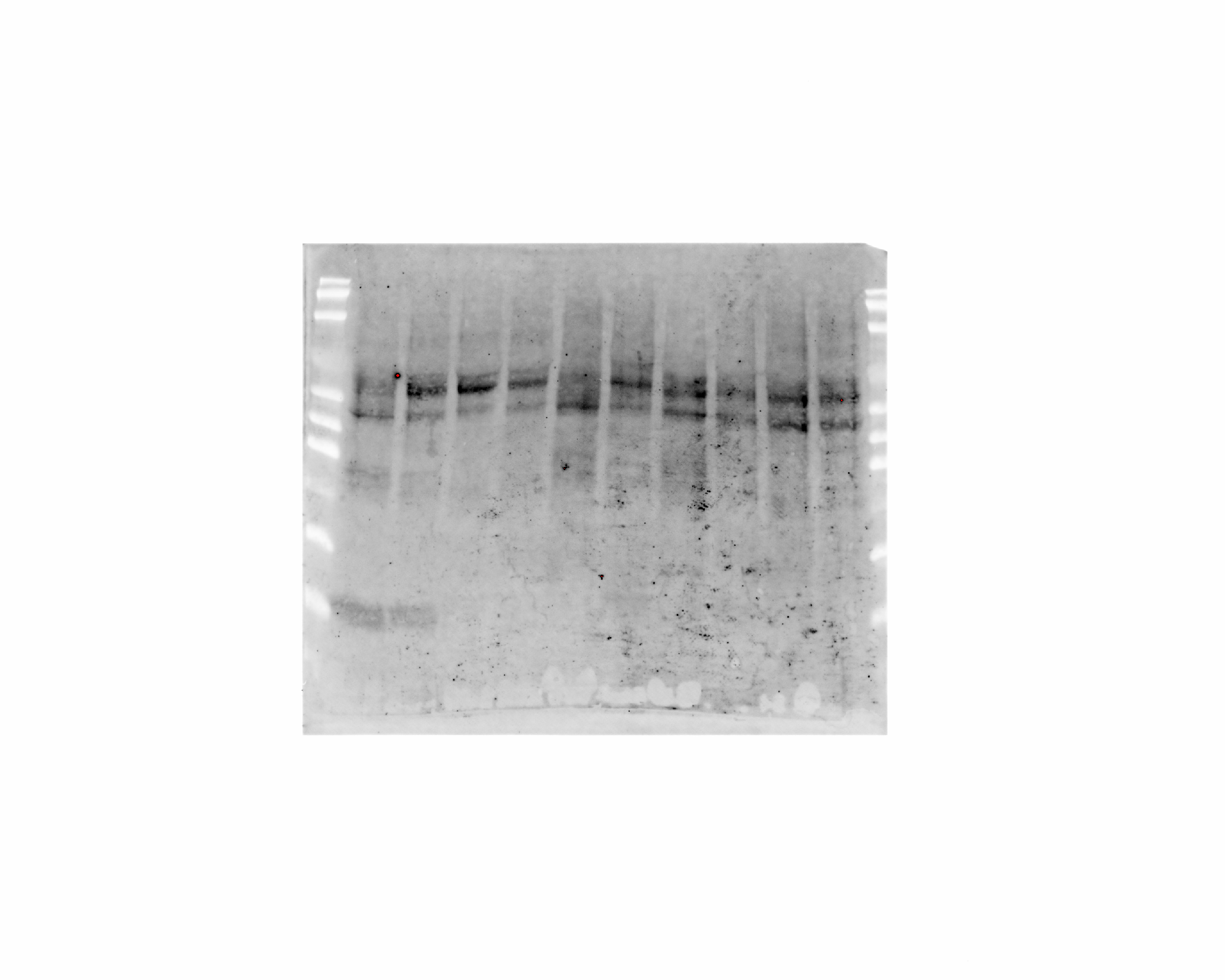

Supplement: Supplementary file 1 [file cancers-16-02726-s001.zip › WB data/Stain Free Blot CD166 PDAC.tif]

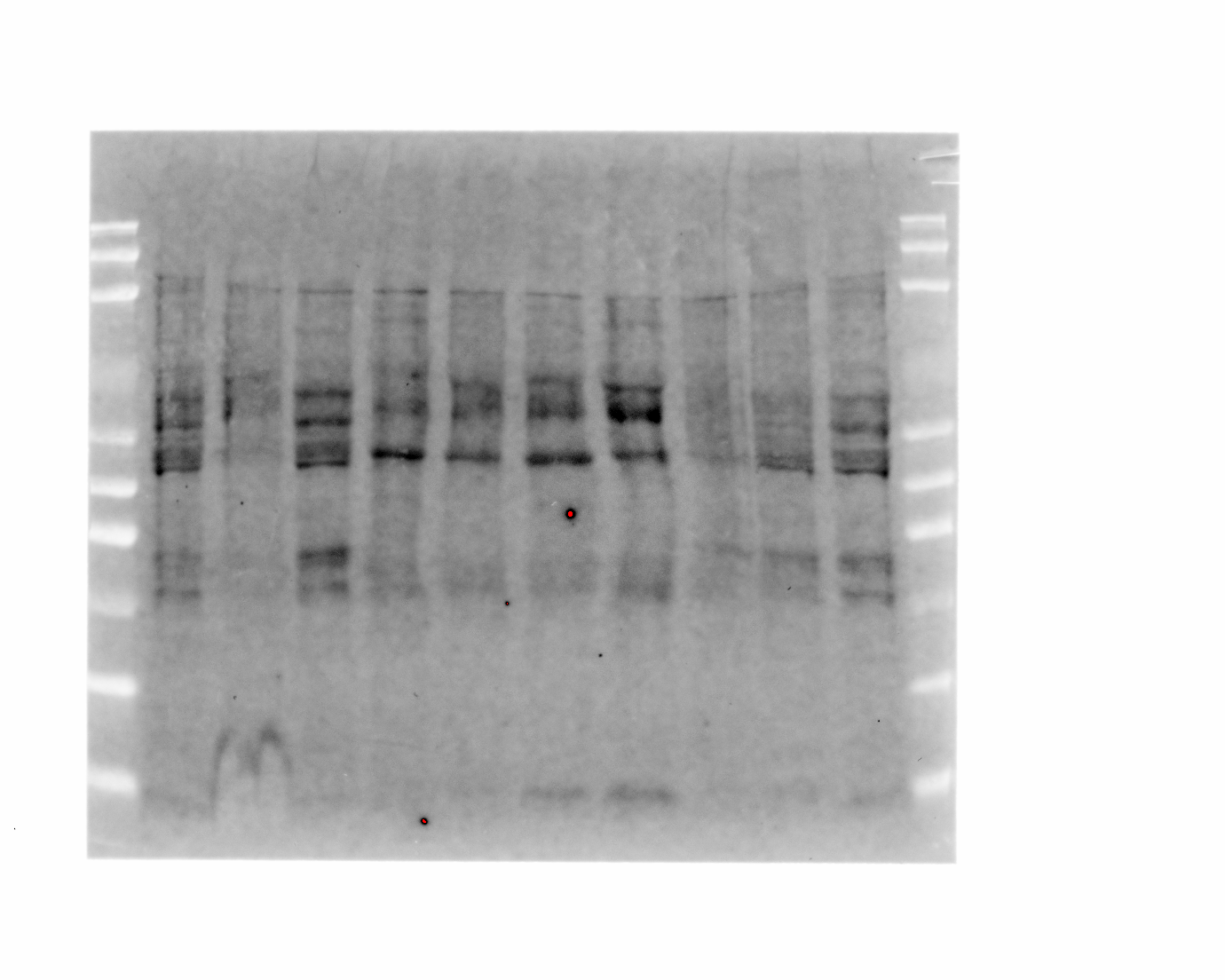

Supplement: Supplementary file 1 [file cancers-16-02726-s001.zip › WB data/Stain Free Blot CD63, CDH1, p53 PDAC+DM.tif]

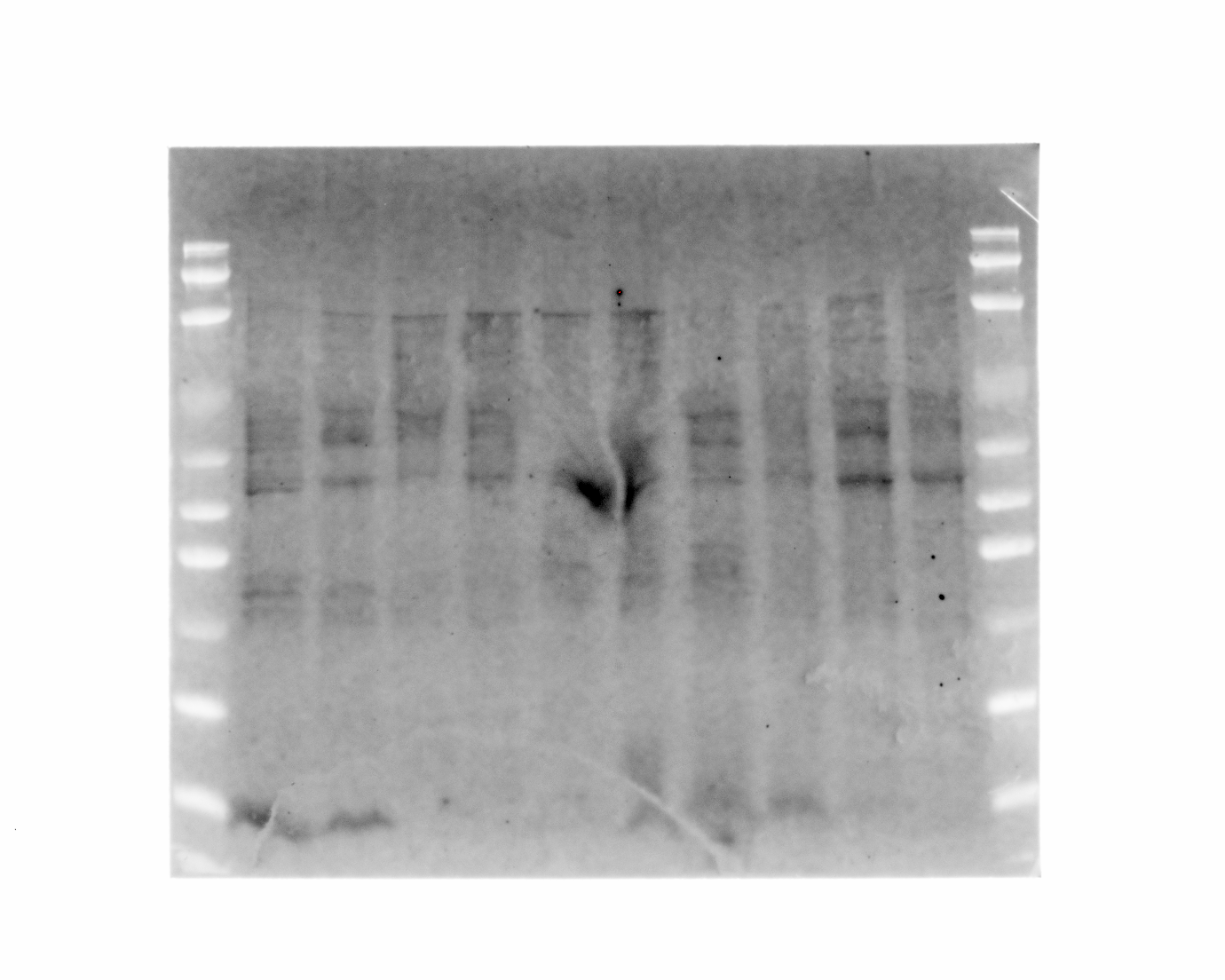

Supplement: Supplementary file 1 [file cancers-16-02726-s001.zip › WB data/stain free blot CD63, CDH1, p53 PDAC.tif]

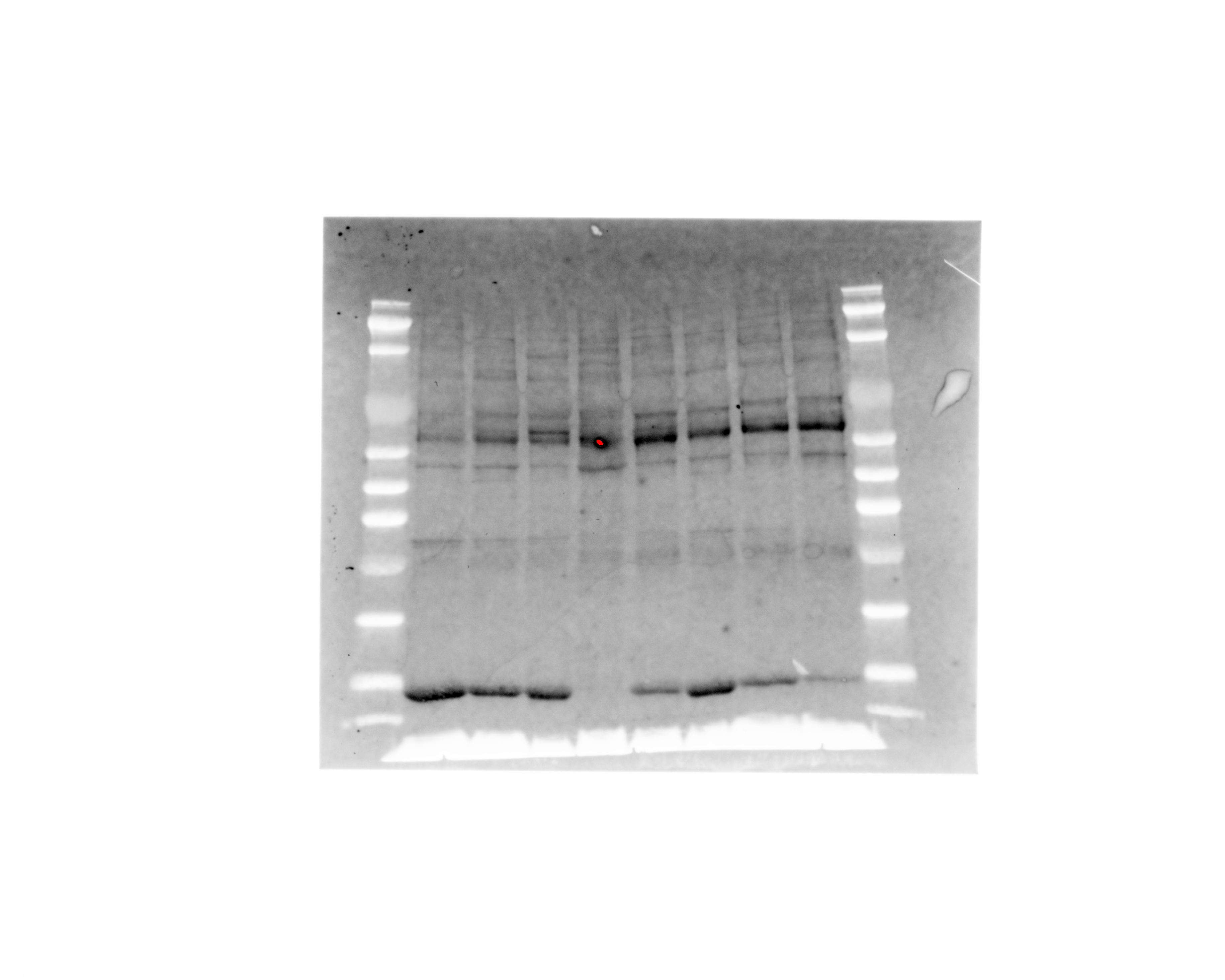

Supplement: Supplementary file 1 [file cancers-16-02726-s001.zip › WB data/stain free blot CDH1, S100 A13 (2), Smad 4 (2) PDAC+DM.tif]

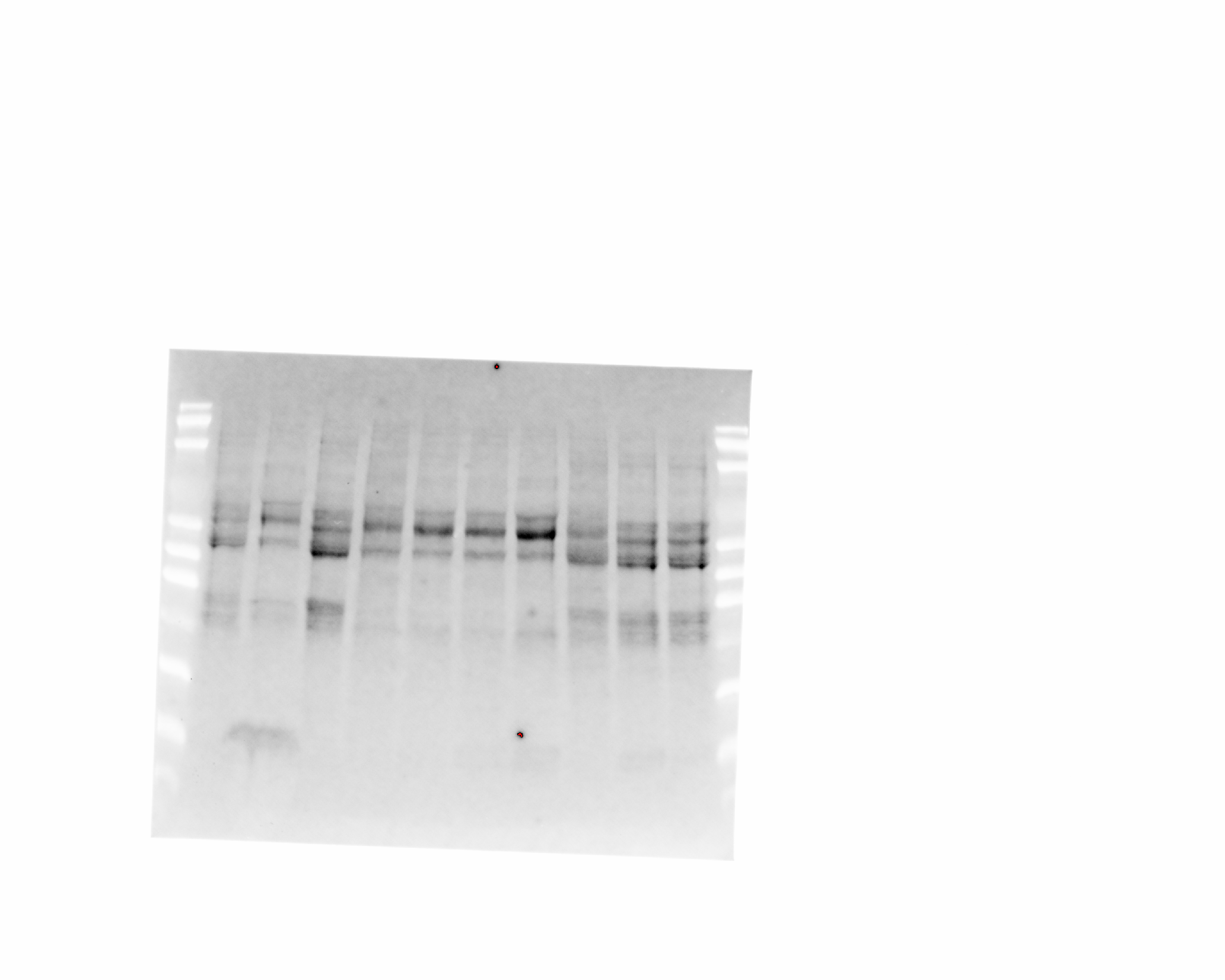

Supplement: Supplementary file 1 [file cancers-16-02726-s001.zip › WB data/Stain Free Blot cJUN, S100 A13 (1) PDAC + DM.tif]

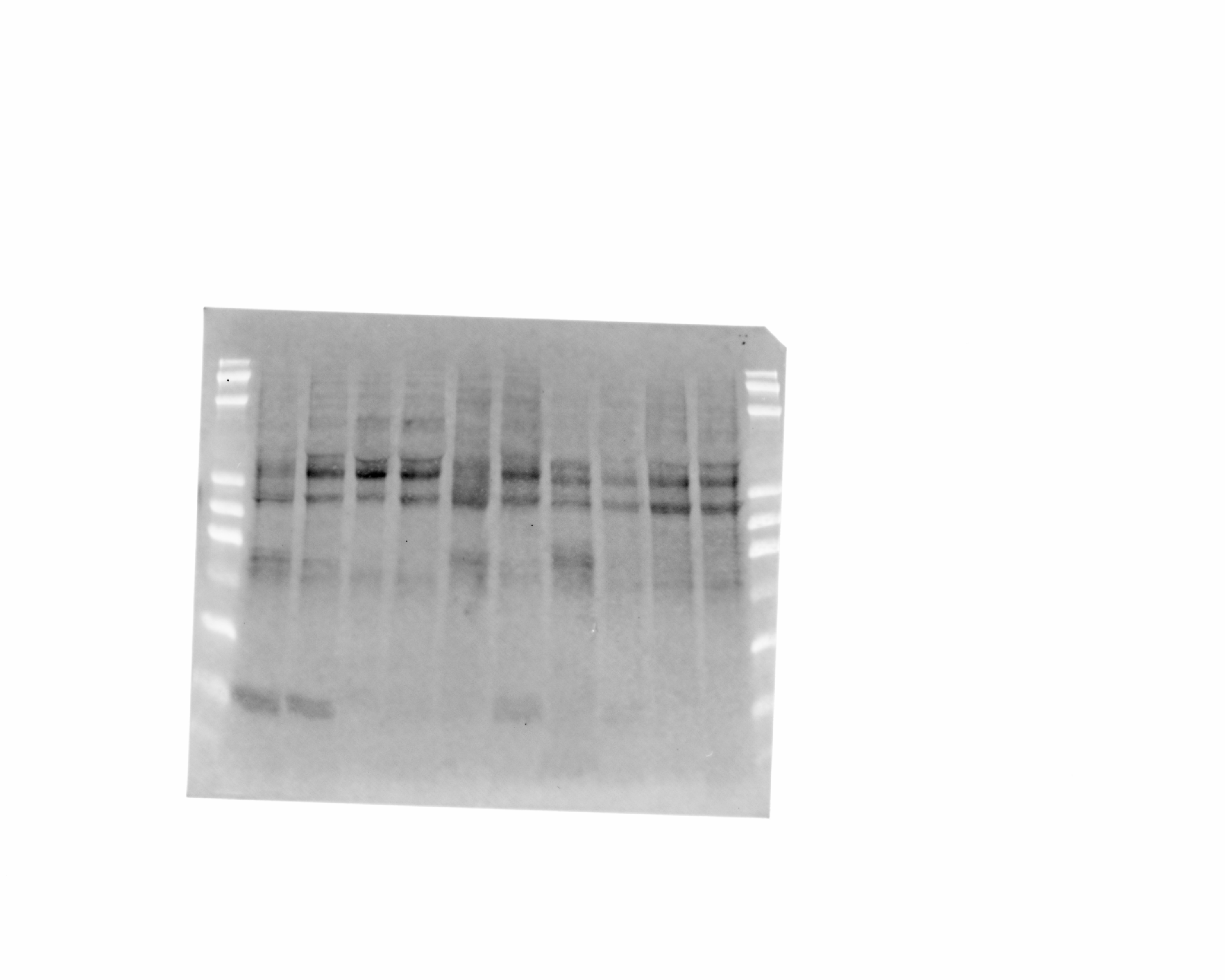

Supplement: Supplementary file 1 [file cancers-16-02726-s001.zip › WB data/Stain Free Blot cJUN, S100A13 PDAC.tif]

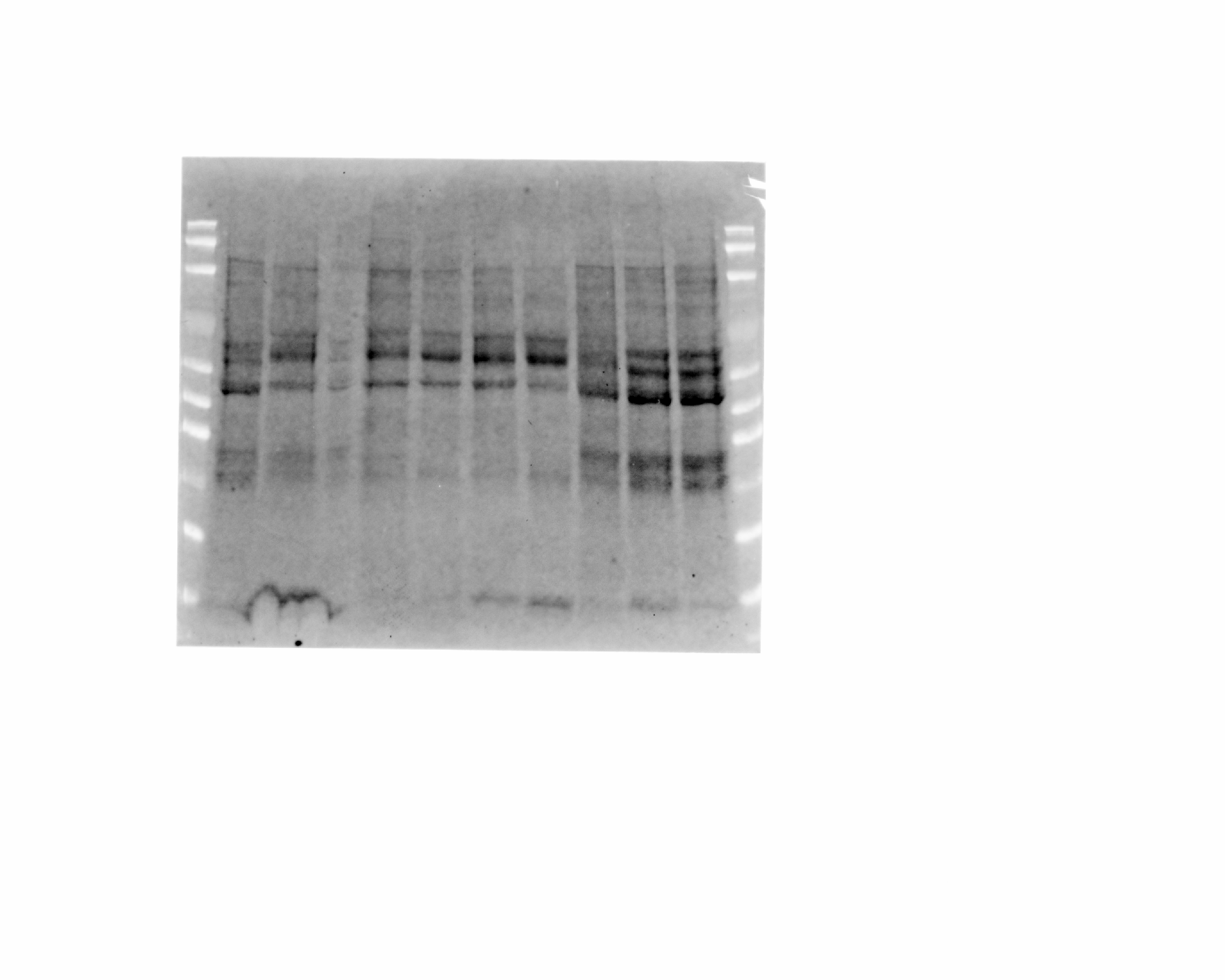

Supplement: Supplementary file 1 [file cancers-16-02726-s001.zip › WB data/Stain Free Blot Smad 4 (1) PDAC+DM.tif]

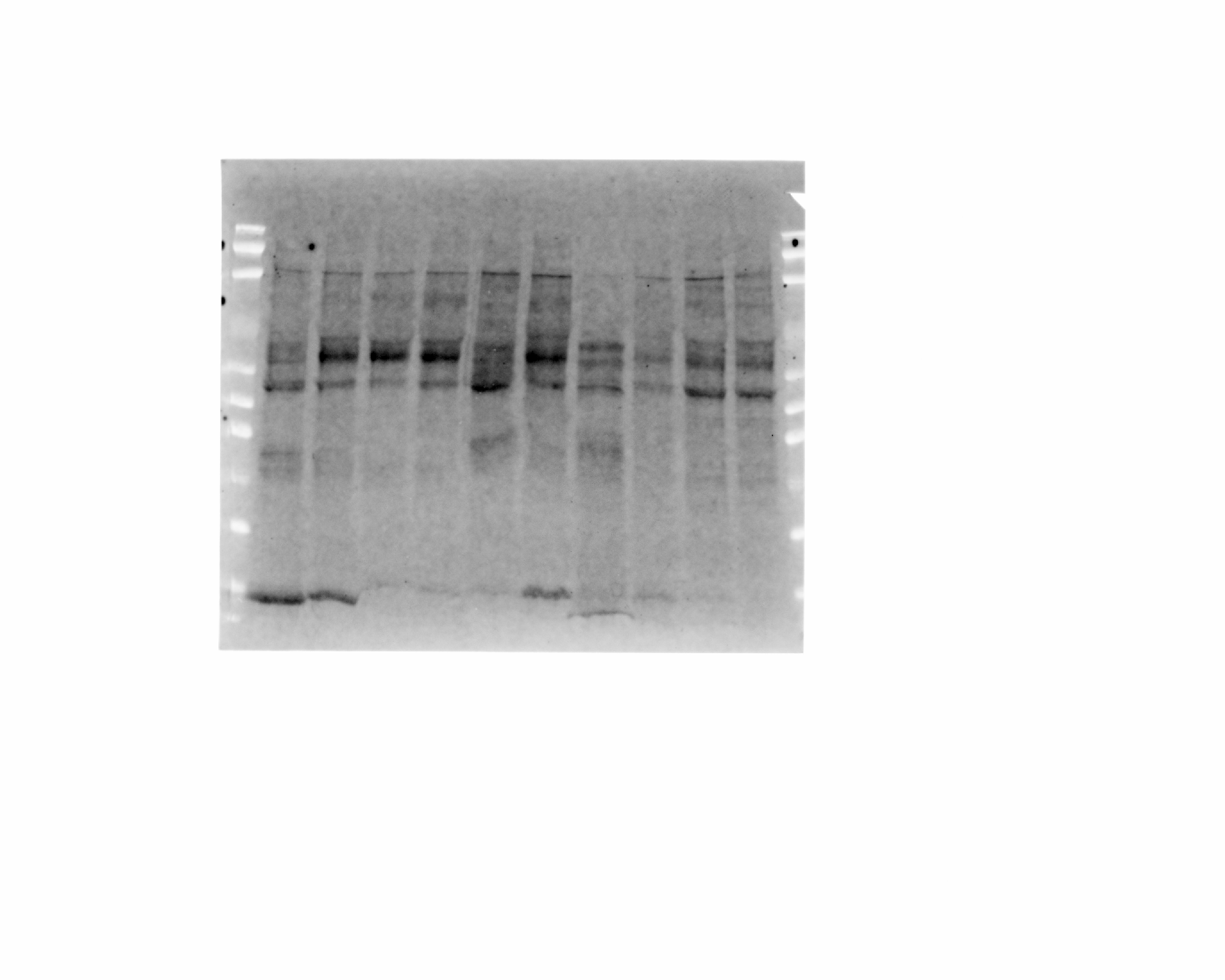

Supplement: Supplementary file 1 [file cancers-16-02726-s001.zip › WB data/Stain Free Blot smad 4 PDAC.tif]

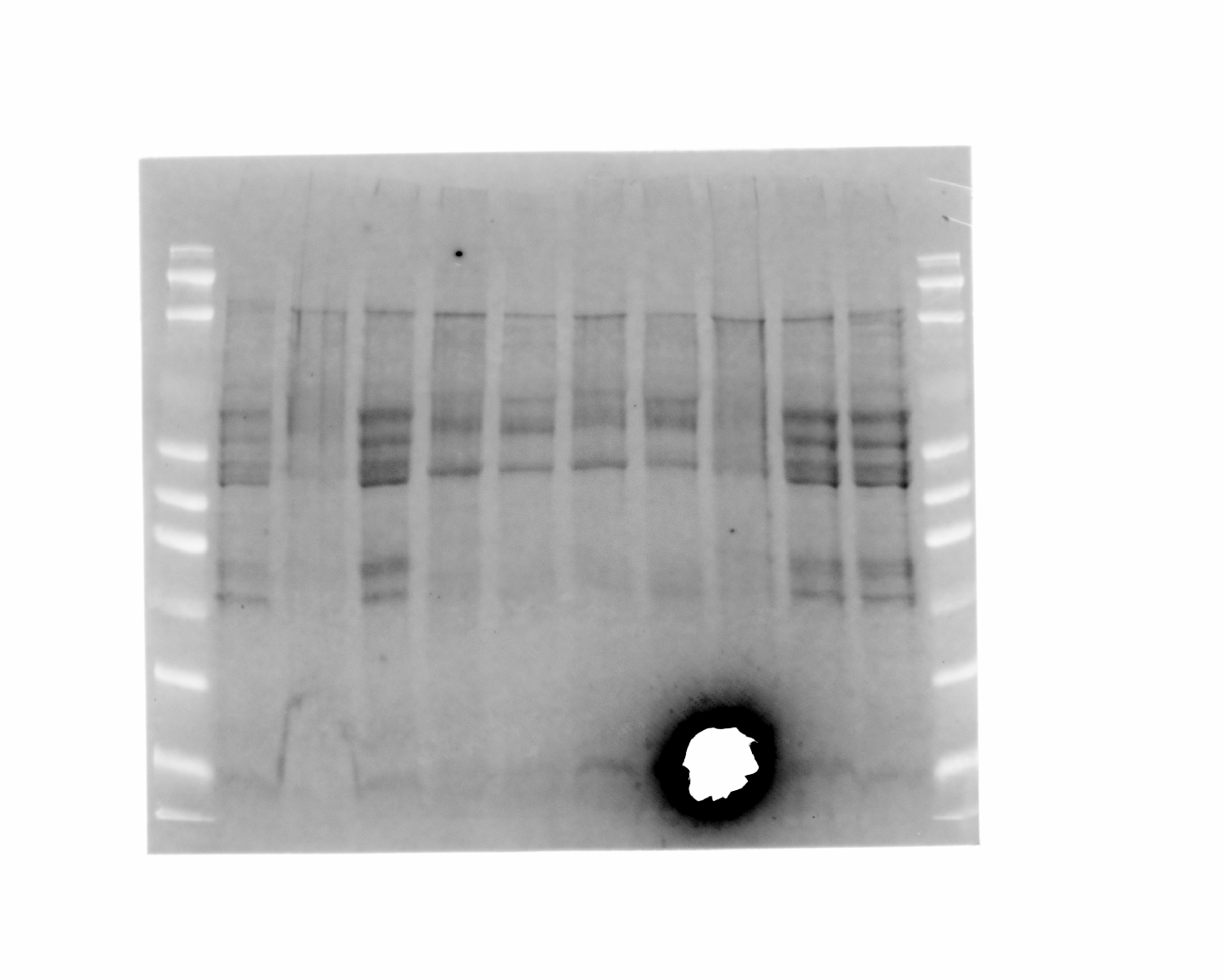

Supplement: Supplementary file 1 [file cancers-16-02726-s001.zip › WB data/Stain Free Blot TGM PDAC +DM.tif]

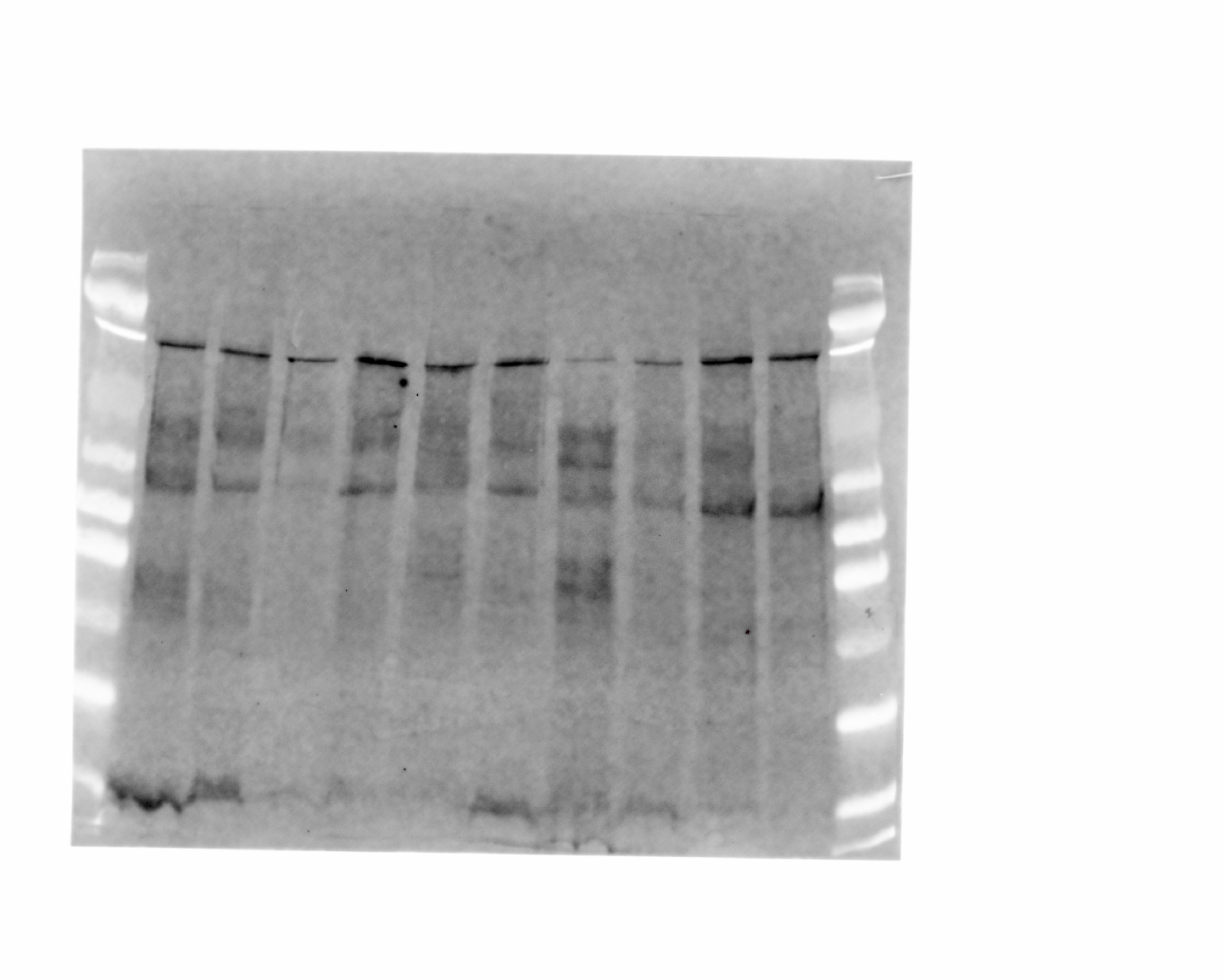

Supplement: Supplementary file 1 [file cancers-16-02726-s001.zip › WB data/Stain Free Blot TGM2 PDAC.tif]

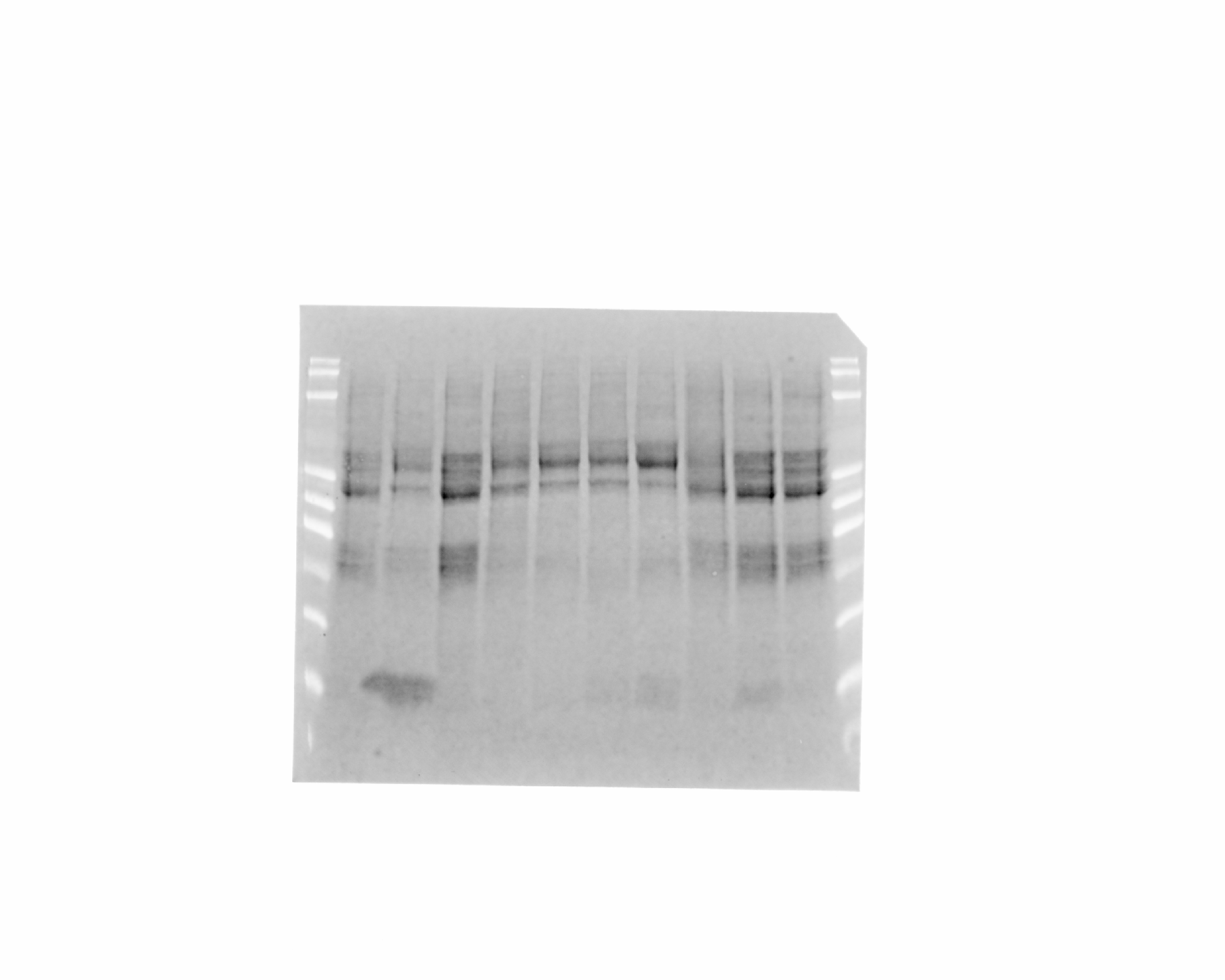

Supplement: Supplementary file 1 [file cancers-16-02726-s001.zip › WB data/Stain Free Blot TNF-_PDAC+DM.tif]

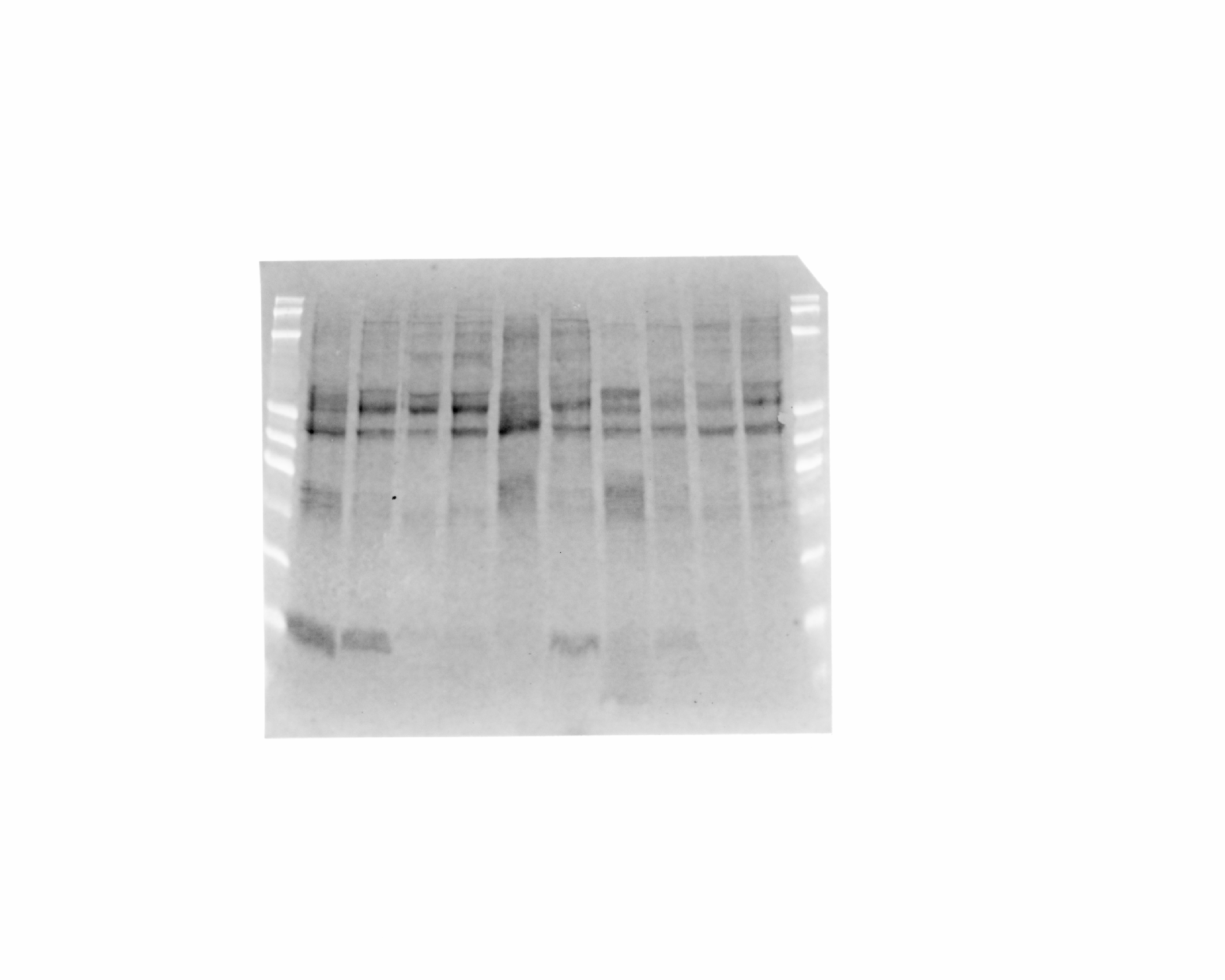

Supplement: Supplementary file 1 [file cancers-16-02726-s001.zip › WB data/Stain Free Blot TNF-_PDAC.tif]

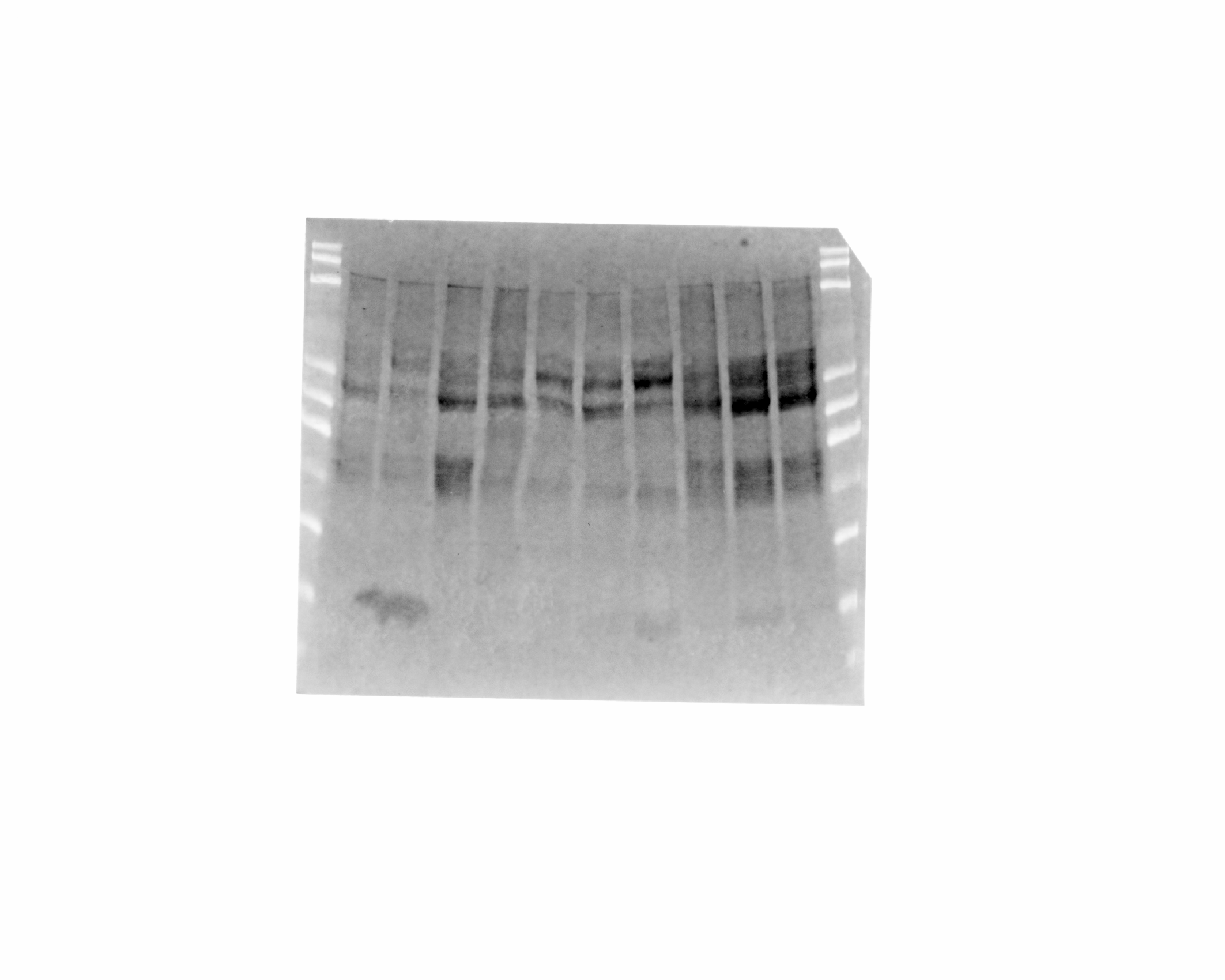

Supplement: Supplementary file 1 [file cancers-16-02726-s001.zip › WB data/Stain Free Blot YAP PDAC+DM.tif]

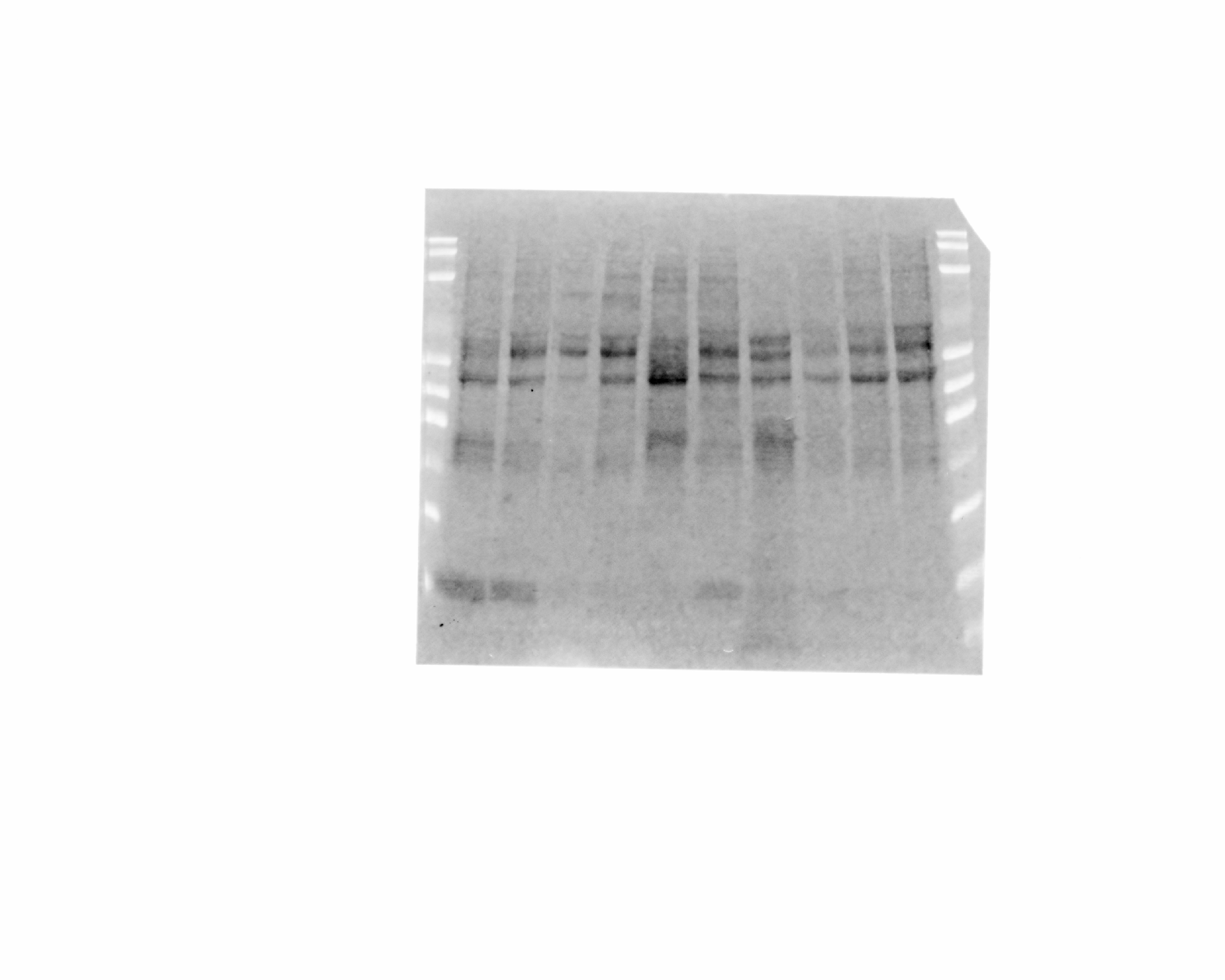

Supplement: Supplementary file 1 [file cancers-16-02726-s001.zip › WB data/Stain Free Blot YAP PDAC.tif]

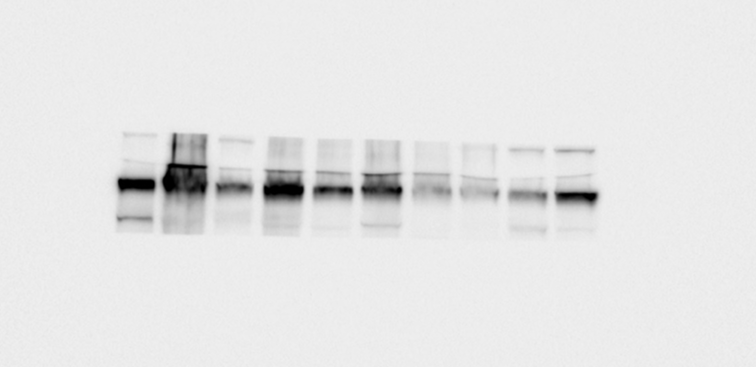

Supplement: Supplementary file 1 [file cancers-16-02726-s001.zip › WB data/TGM2 PDAC +DM.tif]

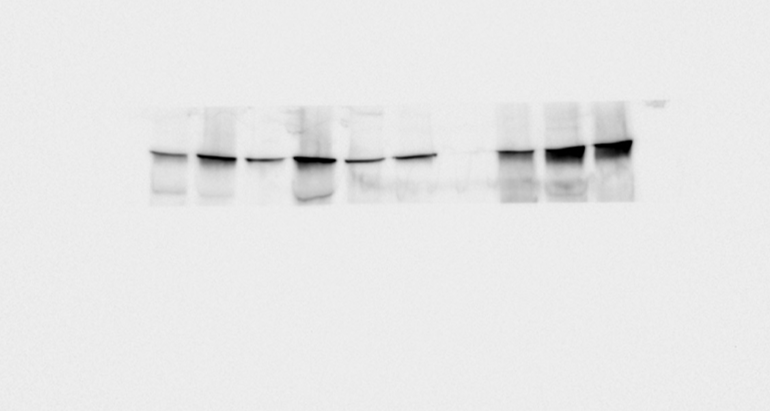

Supplement: Supplementary file 1 [file cancers-16-02726-s001.zip › WB data/TGM2 PDAC.tif]

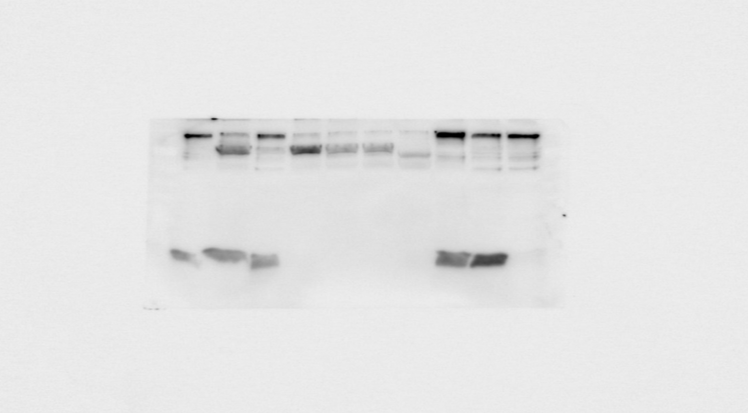

Supplement: Supplementary file 1 [file cancers-16-02726-s001.zip › WB data/TNF _PDAC+DM.tif]

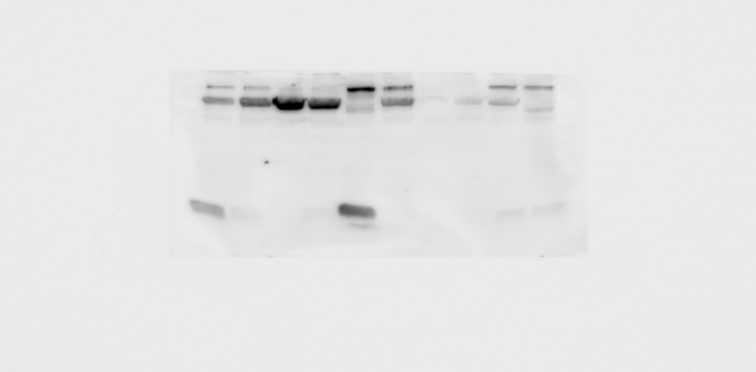

Supplement: Supplementary file 1 [file cancers-16-02726-s001.zip › WB data/TNF _PDAC.tif]

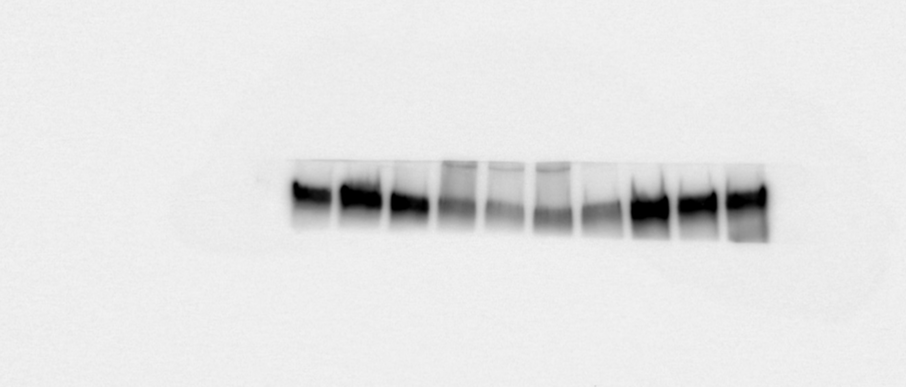

Supplement: Supplementary file 1 [file cancers-16-02726-s001.zip › WB data/YAP PDAC + DM.tif]

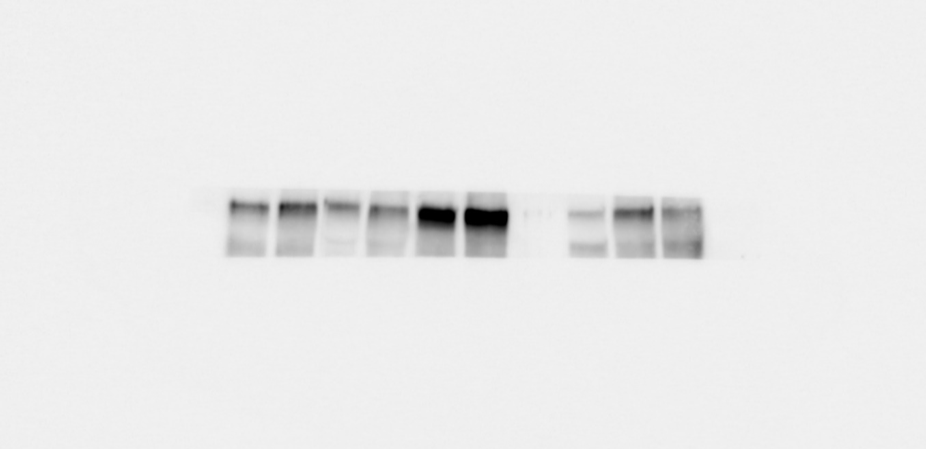

Supplement: Supplementary file 1 [file cancers-16-02726-s001.zip › WB data/YAP PDAC .tif]
